# Computer-aided designed 3D-printed polymeric scaffolds for personalized reconstruction of maxillary and mandibular defects: a proof-of-concept study

**DOI:** 10.1007/s00405-023-08392-0

**Published:** 2024-01-03

**Authors:** Davide Mattavelli, Vincenzo Verzeletti, Alberto Deganello, Antonio Fiorentino, Tommaso Gualtieri, Marco Ferrari, Stefano Taboni, William Anfuso, Marco Ravanelli, Vittorio Rampinelli, Alberto Grammatica, Barbara Buffoli, Roberto Maroldi, Ceretti Elisabetta, Rita Rezzani, Piero Nicolai, Cesare Piazza

**Affiliations:** 1 Unit of Otorhinolaryngology-Head and Neck Surgery, ASST Spedali Civili of Brescia, Brescia, Italy; 2 Department of Medical and Surgical Specialties, Radiological Sciences, and Public Health, University of Brescia, School of Medicine, Brescia, Italy; 3 Thoracic Surgery Unit, Department of Cardiac, Thoracic, Vascular Sciences and Public Health, University of Padua-Azienda Ospedale Università di Padova, Padua, Italy; 4 Otolaryngology Head and Neck Surgery Department of IRCCS, National Cancer Institute (INT), Milan, Italy; 5 Department of Mechanical and Industrial Engineering, University of Brescia, Brescia, Italy; 6 Department of Otorhinolaryngology, Head and Neck Surgery, “Nuovo Santo Stefano” Civil Hospital, Prato, Italy; 7 Unit of Otorhinolaryngology-Head and Neck Surgery, Department of Neurosciences, University of Padua-Azienda Ospedale Università di Padova, Padua, Italy; 8 Guided Therapeutics (GTx) Program International Scholarship, University Health Network (UHN), Toronto, ON Canada; 9 Artificial Intelligence in Medicine and Innovation in Clinical Research and Methodology (PhD Program), Department of Clinical and Experimental Sciences, University of Brescia, Brescia, Italy; 10 Unit of Radiology, ASST Spedali Civili of Brescia, Brescia, Italy; 11 Section of Anatomy and Physiopathology, Department of Clinical and Experimental Sciences, University of Brescia, School of Medicine, Brescia, Italy

**Keywords:** Computer-aided design, 3D printing, Maxillofacial reconstruction, Scaffold, Maxillary, Mandible

## Abstract

**Purpose:**

To investigate the potential reconstruction of complex maxillofacial defects using computer-aided design 3D-printed polymeric scaffolds by defining the production process, simulating the surgical procedure, and explore the feasibility and reproducibility of the whole algorithm.

**Methods:**

This a preclinical study to investigate feasibility, reproducibility and efficacy of the reconstruction algorithm proposed. It encompassed 3 phases: (1) scaffold production (CAD and 3D-printing in polylactic acid); (2) surgical simulation on cadaver heads (navigation-guided osteotomies and scaffold fixation); (3) assessment of reconstruction (bone and occlusal morphological conformance, symmetry, and mechanical stress tests).

**Results:**

Six cadaver heads were dissected. Six types of defects (3 mandibular and 3 maxillary) with different degree of complexity were tested. In all case the reconstruction algorithm could be successfully completed. Bone morphological conformance was optimal while the occlusal one was slightly higher. Mechanical stress tests were good (mean value, 318.6 and 286.4 N for maxillary and mandibular defects, respectively).

**Conclusions:**

Our reconstructive algorithm was feasible and reproducible in a preclinical setting. Functional and aesthetic outcomes were satisfactory independently of the complexity of the defect.

**Supplementary Information:**

The online version contains supplementary material available at 10.1007/s00405-023-08392-0.

## Introduction

Reconstruction of bone-including defects of the craniofacial skeleton is challenging in view of several issues. The reconstruction must provide an adequate mechanical support, preserve basic physiological functions, such as breathing, swallowing, binocular view, and guarantee an acceptable aesthetic outcome. Currently, re-vascularized bone-containing free flaps represent the gold standard to meet these needs [1–3]. However, these reconstructions are technically demanding and require high expertise. Moreover, donor site is a further source of complication and its morbidity can be non-negligible even for expert surgeons, thus limiting indications in unfit patients [4–6].

Over the last decades, research has made much progress in the field of tissue bioengineering to create bony, cartilaginous, and epithelial tissues. Scaffolding is a branch of tissue bioengineering that uses “framework” materials which are capable of hosting tissue formation. These innovations in materials, design, and fabrication processes have allowed the possibility to produce devices with high performance. The fabrication of three-dimensional (3D) polymeric scaffolds of high geometrical complexity can be accomplished through computer-aided design (CAD) and additive manufacturing (AM) technologies, which allow realizing 3D parts by merging layers of material one over the other [7–10]. These technologies may represent a valid solution for producing aim-specific, customized objects in a relatively short time span. Moreover, polymers may offer the possibility of fine-tuning the mechanical properties and time of resorption of the scaffold.

Definition of an efficient production algorithm and precise planning of the surgical procedure are essential to fully exploit the potential of these techniques. As the reconstruction usually follows the ablative phase in a single-stage procedure, the production process of the scaffold needs to predict the extension of the defect; in turn, bony resection must follow the planned cutting lines to optimize the bone-scaffold interface and ease plug fixation. For complex maxillary and mandibular defects, high-quality data simulating the entire workflow in a preclinical, controlled setting are currently lacking and are mandatory for future clinical applications.

The present work is a preclinical proof-of-concept study to investigate the potential reconstruction of extensive and complex maxillary and mandibular defects using CAD 3D-printed polymeric scaffolds. It aims at defining the production process of the scaffold, simulating the surgical procedure for the maxillofacial reconstruction, and explore the overall feasibility and reproducibility of the whole algorithm. It is intended as a first step of a wider research line that will explore the bioengineering of the scaffold with human mesenchymal stem cells (hMSC), where the re-adsorbable scaffold should be timely replaced by newborn bone.

## Methods

### Study objectives

The primary objective of the study is to define the production process and verify its feasibility and reproducibility by simulating different scenarios of maxillofacial reconstructive surgery on cadaver heads.

Secondary objectives are: (a) verifying the accuracy of the reconstruction by comparing its morphological contour with the original one (bone morphological conformance); (b) evaluating the aesthetic outcome thanks to symmetry analysis with the contralateral side; (c) testing the efficiency and mechanical resistance of the reconstruction thanks to occlusal force tests; (d) conducting an exploratory test to include dental restoration into scaffold planning and printing.

### Study plan

**Fig. 1:**
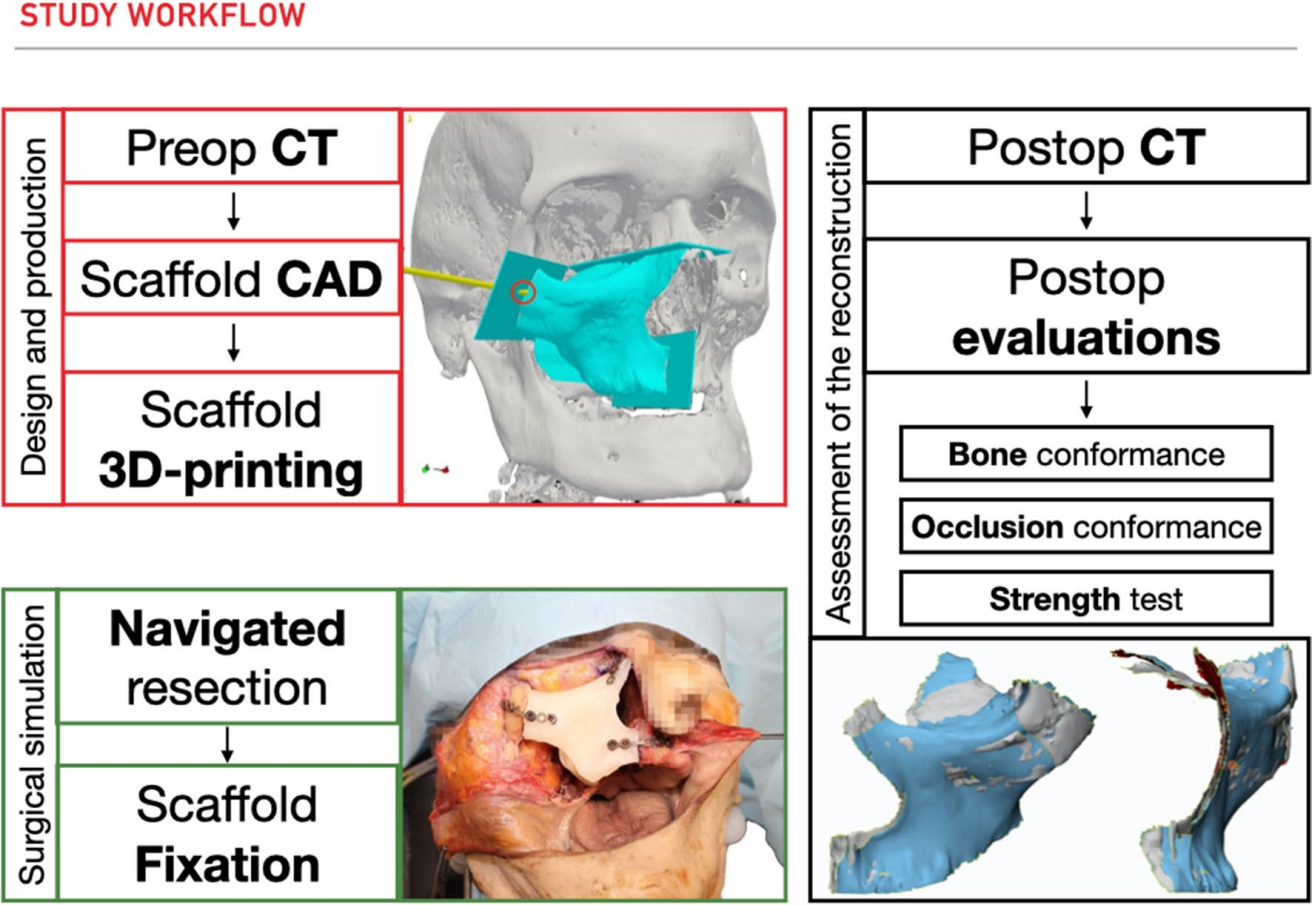
A schematic overview on the study workflow

The workflow was divided into 3 subtasks: (1) scaffold design and production; (2) simulation of the surgical procedure in a preclinical setting; (3) assessment of the reconstruction (Fig. 1).

We studied 6 types of bony defects (3 mandibular and 3 maxillary) with an increasing degree of complexity.

All maxillary defects had the medial bony cut on the midline of the hard palate, while they had a different vertical extension [11]:

1. type I: inferior maxillectomy, with the superior cut just above the maxillary floor (preserving the anterior maxillary wall);
2. type II: subtotal maxillectomy, with the superior cut just below the inferior orbital rim (preserving the orbital floor);
3. type III: total maxillectomy, including the orbital floor.

For mandibular defects we referred to the classification proposed by Brown et al. [12]:

4. type I: lateral defect not including ipsilateral canine or condyle;
5. type II: hemi-mandibulectomy including ipsilateral, but not contralateral canine or condyle;
6. type III: anterior mandibulectomy including both canines, but neither angle.

### Scaffold design and production

**Fig. 2:**
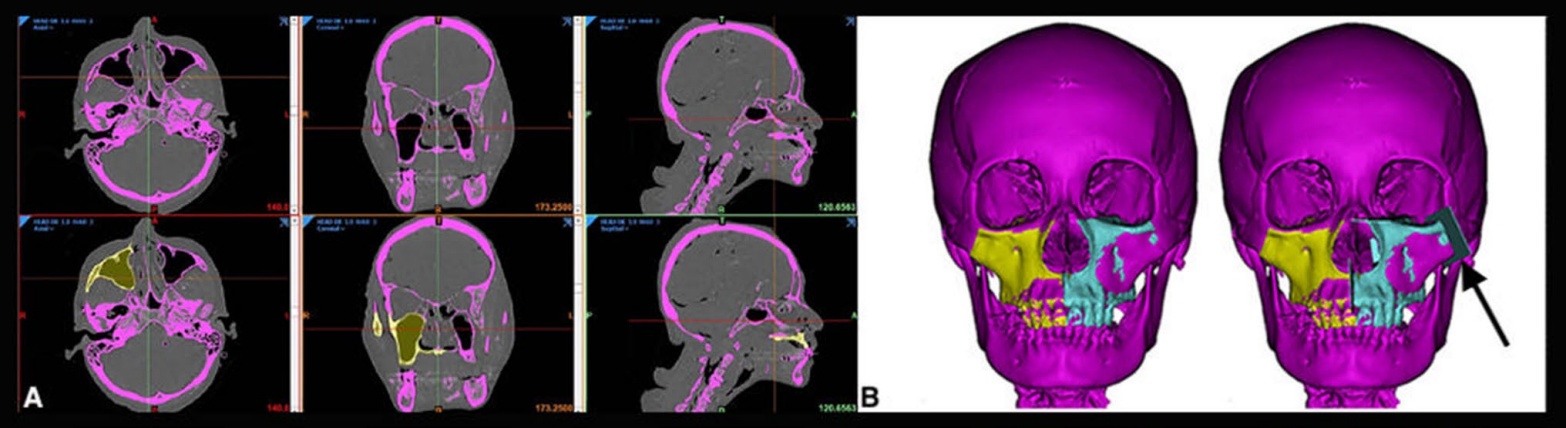
The segmentation processes. **A** Semi-automatic segmentation of the selective mask for the bone tissue (purple mask), the specific mask for the maxillary region (yellow mask); **B** creation of the matching 3D solid (yellow solid) and its contralateral transposition (light blue) with subsequent generation of the cutting lines (black arrow)

Computed tomography (CT) DICOM images were obtained for each cadaver head with the multidetector 128-slice CT scanner Somatom Definition Flash^®^ (Siemens AG, Munich, DE) with 0.7 mm axial slices and were subsequently uploaded to Materialise Mimics^®^, version 21 (Materialise NV, Leuven, BE). The region of interest was segmented and the matching solid obtained was 'mirrored' on the contralateral side (Fig. 2). This step was meant to simulate the clinical condition where the ipsilateral bony anatomy is altered by the tumor, and the scaffold must be designed on the contralateral side. It was not performed for median defects (i.e., Brown type III). When present, the segmentation process also included teeth.

Next, cutting lines were created according to the abovementioned boundaries of the defects. The designed scaffold was exported in two*.stl* format files: one with cutting lines to be uploaded in the navigation system; the other without, for post-segmentation modifications (Meshmixer^®^, version 3.5, Autodesk Inc, Mill Valley, US-CA and Solidworks^®^, version 2019 Dessault Systèmes, Vélizy-Villacoublay, FR). Post-segmentation modifications depended on the type of the defect and included smoothening of the outer surfaces to ease printing and avoid injury to soft tissues, smooth cavitation of the maxillary scaffold (that was previously designed as a full solid), planning of holes for screws, and adding supports for scaffold fixation. The latter were tentatively applied only to mandibular scaffolds and were constituted by extensions of the inferior border of the scaffolds that were able to cover and adapt to the inferior border of the native mandible; they also had holes where the screws could be driven to fix the reconstruction (Supplementary Fig. 1). The designing process was finally checked through Gom Inspect^®^ (Zeiss, Jena, GE) to evaluate differences between the*.stl* file before and after the post-segmentation refinements; spatial discrepancies had to be < 1 mm.

The plug was printed in polylactic acid (PLA) (Pearl White PLA, Ø 2.85 mm, Ultimaker, Utrecht, NL) using Fused Filament Fabrication (FFF) technology with the 3D-printer Ultimaker 3 Extended^®^ (Ultimaker, Utrecht, NL). For each defect, 2 scaffolds were printed (one with the screw holes and one as a rescue option); an additional plug with supports for scaffold fixation was printed for mandibular defects (with supports for scaffold fixation).

### Simulation of the surgical procedure in a preclinical setting

Six fresh frozen cadaver heads (MedCure Inc, Portland, Oregon, USA) were dissected at the Dissection Laboratory of the University of Brescia, School of Medicine. The specimens (4 females, 2 males, mean age at death 71 years) had the arterial system injected with a bi-component red silicon (Down Corning, Midland, US-MI). In each cadaver head two defects were created on opposite sides (1 maxillary and 1 mandibular); therefore, each defect was tested twice. For occlusion analysis, the gingival-dental cast (Lascod S.P.A, Firenze, IT) of the upper and lower maxilla was taken before and after the procedure.

For maxillary resection, a Weber Ferguson lateral nasal skin incision with lip split was performed and a cheek flap was elevated to fully expose the bone. For mandibular resection, a cervical skin incision was performed and a visor flap was elevated without splitting the lip. Incisions and flap elevation were adapted according to the planned defect.

**Fig. 3:**
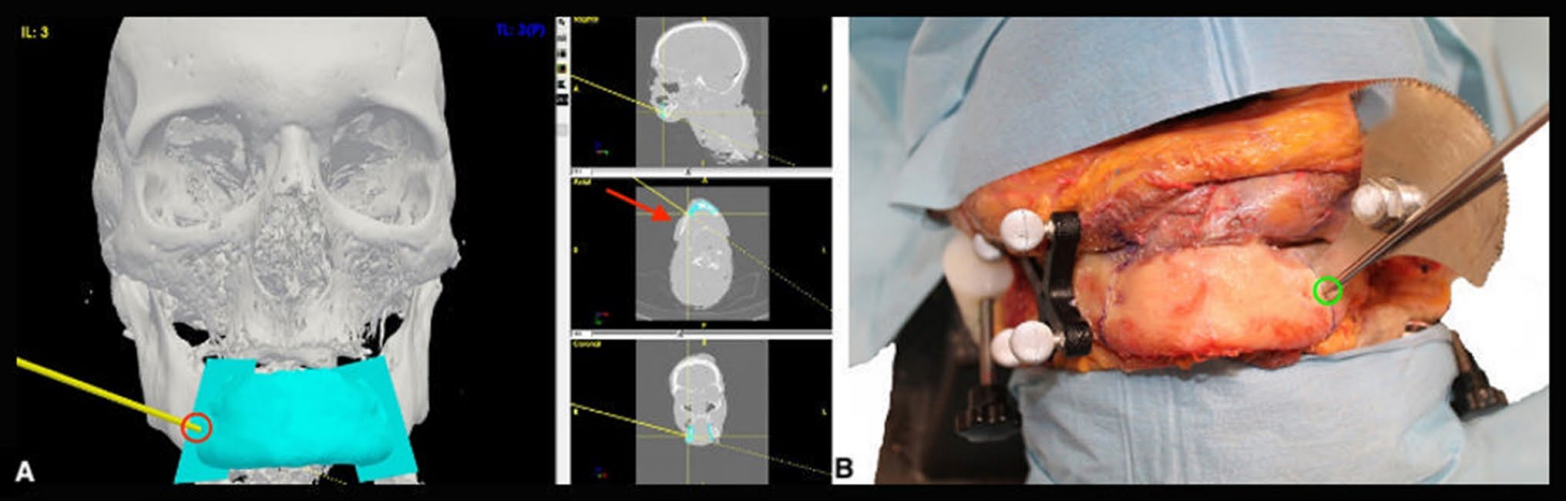
Navigation-guided osteotomies. The positioning of the pointer (**A** red circle and **B** green circle) close to the saw allows obtaining the correct inclination of the osteotomy (red arrow)

**Fig. 4:**
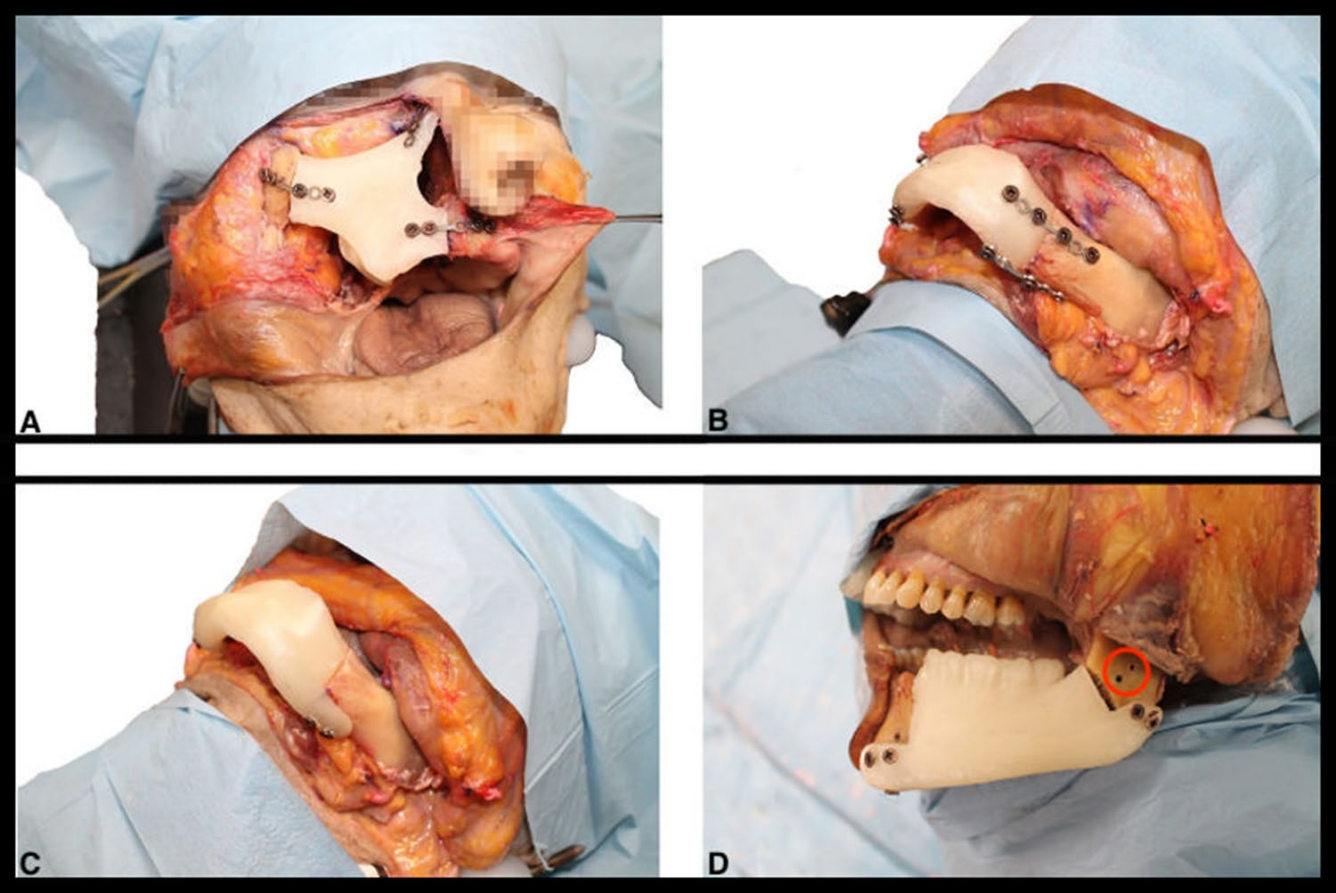
Fixation of four PLA 3D-printed scaffolds with screw and plates. **A** Fixation with 3 plates of a maxillary scaffold after a type III maxillectomy; **B** fixation with 4 plates of a mandibular scaffold after a type III mandibulectomy; **C** fixation of a scaffold with integrated supports after a type III mandibulectomy; **D** fixation of a scaffold with integrated supports after a type I mandibulectomy. The red circle shows the two holes used to position the scaffold devoid of the integrated supports.

The extent of the resection was navigation-guided [13]. CT DICOM images with the designed scaffold and predicted cutting planes were uploaded to the Polaris Vicra^®^ optical navigation system (Northern Digital Incorporated, Waterloo, CA). Thanks to dedicated software (GTx-Eyes 3D navigation system^®^, University Health Network, Toronto, CA), it was possible to navigate the CT on the surgical specimen and create the bony defect as planned and check the correct inclination of the saw, which is paramount to optimize the bone-scaffold interface (Fig. 3 and Supplementary Fig. 2). After this procedure, the scaffold was placed to fill the bony defect and fixed with 2.5 mm-diameter screws of different length (5–8–25 mm) and titanium plates (length × thickness: 31.5 mm × 1.5 mm; 26.2 mm × 1.5 mm; 31.2 mm × 1 mm). To test which scaffold worked best, the reconstruction was accomplished using first the scaffold with pre-designed holes for fixation with plates and screws. For the mandibular defects, the reconstruction was also repeated using the plug with supports for fixation (Fig. 4).

### Assessment of the reconstruction

The quality and robustness of the reconstruction was tested through 3 different functional and quantitative tests.

The first parameter was the bone morphological conformance. The scaffold was segmented on postoperative CT DICOM images, and the surfaces of the native bone and the scaffold were superimposed and compared using Materialise 3-Matic^®^ software (Materialise NV, Leuven, BE). The measure of the mean morphological difference between the two surfaces was calculated through the root mean square deviation (RMS, Supplementary Fig. 3). This parameter has been used in different studies to compare the morphological conformance of maxillofacial reconstructions, and RMS less than 3 mm was considered good [14–18]. We also performed symmetry analysis by comparing the defect side with the contralateral one. This was considered an aesthetic outcome.

The second was the occlusal morphological conformance: dental impressions were cast into a 3D plaster model, which was digitalized with a 3D-scanner. The*.stl* files were processed as previously described, and the difference between the two dentition sets was expressed as RMS.

**Fig. 5:**
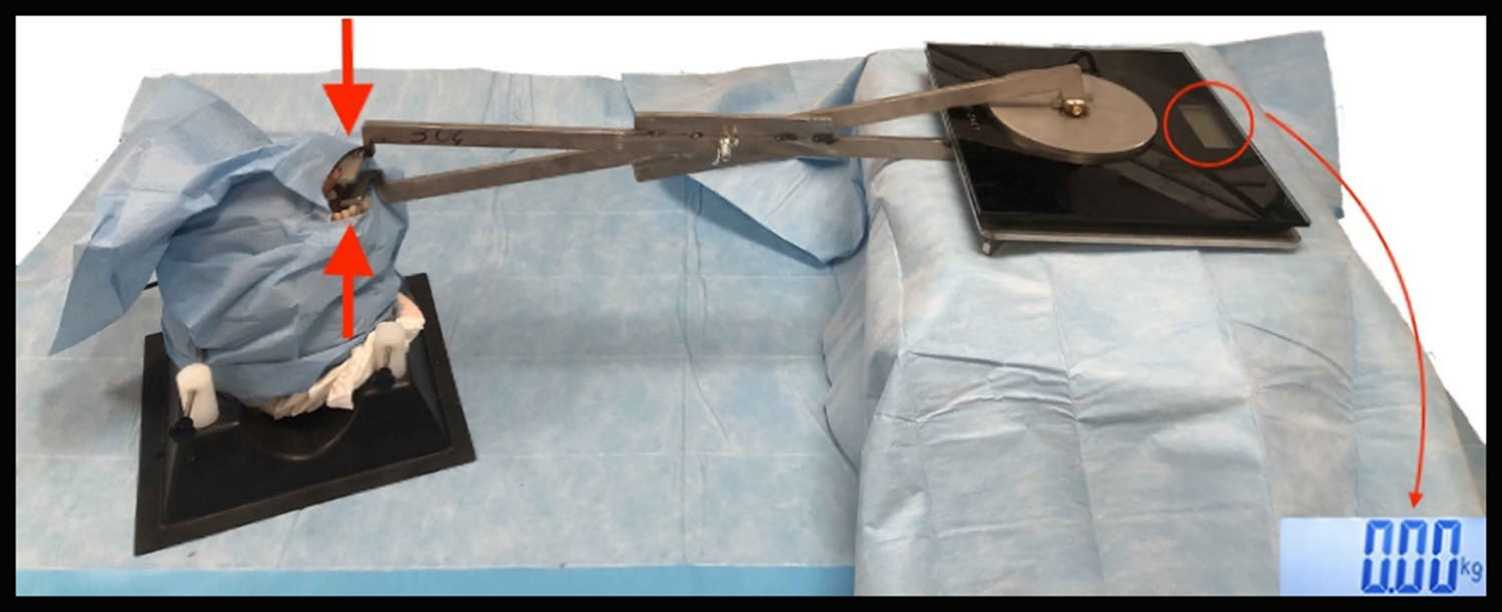
Illustration of the instrument used for mechanical stress tests (*N* = kg × 9.8)

The third test was a mechanical stress test to quantify the maximal bite force carried by the reconstruction until rupture of the plug or surrounding bones. For this purpose, we created a dedicated device that can measure the mechanical resistance of the scaffold with respect to a purely vertical force component generated by the operator closing the bite (Fig. 5). The tool was designed to test the vertical force generated on a single side of the oral cavity, so that the two reconstructions in the same cadaver head could be tested separately. For mandibular defects, the test was performed twice since two different types of scaffolds were used, and the fixation with screws and plates was always tested first. Subjective considerations of the operators were also annotated.

## Results

### Scaffold design and production

The production process required on average 40 h (range 36–44 h). In addition, the scaffold had to be soaked in water for 24 h to eliminate any traces of soluble components from the PLA printed device.

Overall, 30 scaffolds were printed: 12 for maxillary defects (6 with holes and 6 without), and 18 for mandibular defects (as above with the addition of 6 plugs with integrated supports for fixation). In all cases, dimensional gaps between the*.stl* file generated by the segmentation process and the*.stl* file obtained after post-segmentation modification were < 1 mm.

### Simulation of the surgical procedure in a preclinical setting

Twelve procedures (6 maxillectomies and 6 mandibulectomies) were performed on 6 cadaver heads (2 per head, 1 per side).

A navigation system was effectively set up in all cases. The navigation error was always < 1 mm, (mean: 0.65 mm; median: 0.62 mm). This accuracy was constant in both maxillectomies (mean: 0.69 mm; median: 0.65 mm) and mandibulectomies (mean: 0.60 mm; median: 0.54 mm). As per surgeon’s reports, positioning of the scaffold was always straightforward with an excellent bony-plug interface. In no case were adjustments of the bony edges deemed necessary after osteotomies were performed.

Scaffold fixation was efficient using screws and plates, and screws and PLA supports for mandibular plugs. In all cases fixation could be achieved with the scaffold with pre-designed holes for screws, and no drilling of new holes was required. In type III maxillary defects, the scaffold was further stabilized via a 25 mm-long screw driven in the pterygoid root under endoscopic guidance.

In relation to the mandibular scaffolds equipped with PLA supports, fixation was effective in all 6 cases and in no case did the support break during driving of the screws.

The procedure of scaffold placement and fixation was time saving. Overall, the mean time to fix the scaffold with plates and screws in all 12 defects was 50 min. The average time did not differ significantly between maxillary and mandibular plugs (51.6 and 48.3 min, respectively) with similar ranges of variations according to the complexity of the defect (40–60 and 35–55 min, respectively). Conversely, scaffolds with support for fixation (only for mandibular defects) were much quicker to fix and required only 14.5 min on average.

Maxillary scaffolds were tentatively resurfaced with vascularized tissue using temporo-parietal fascial flap or palatal mucosal island flap (oral side) and a naso-septal flap (nasal side). Coverage was overall satisfactory.

### Assessment of the reconstruction

Data on bone morphological conformance, symmetry analysis, occlusal morphological conformance and strength tests detailed according to the defect and the single test are reported in Table 1.

**Table 1:** Assessment of the reconstruction according to the specific defect type

Maxillary DEFECT	RMS bone conformance (mm)			DELTA symmetry (mm) (Sym POST-Sym PRE)			RMS Occlusion (mm)			Strength test (N)		
	Test 1	Test 2	Mean	Test 1	Test 2	Mean	Test 1	Test 2	Mean	Test 1	Test 2	Mean
Type I	0.81	2.58	1.70	+ 0.58	+ 0.23	+ 0.40	1.14	1.87	1.50	411	267	339
Type II	1.12	1.14	1.13	+ 0.04	+ 0.01	+ 0.02	1.10	2.06	1.58	205	392	298.50
Type III	1.26	1.72	1.49	+ 0.62	+ 0.44	+ 0.53	0.91	2.85	1.88	257	380	318.50

Mandibular DEFECT	RMS bone conformance (mm)			DELTA symmetry (mm) (Sym POST-Sym PRE)			RMS Occlusion (mm)			Strength test (N)		
	Test 1	Test 2	Mean	Test 1	Test 2	Mean	Test 1	Test 2	Mean	Test 1	Test 2	Mean
Type I	1.28	1.97	1.63	+ 2.29	+ 0.78	+ 1.53	1.68	1.52	1.60	429	304.70	366.85
Type II	1.03	1.83	1.43	− 0.05	+ 0.74	+ 0.34	1.31	1.61	1.46	218	179.30	198.65
Type III	0.73	1.14	0.93	+ 0.34	− 0.06	+ 0.14	1.14	1.14	1.14	205	380	292.50

#### Bone morphological conformance

The RMS ranged between 0.73 mm (type III mandibular defect) and 2.58 mm (type I maxillary defect) with a mean value of 1.38 mm.

RMS was slightly higher in the maxillary model (mean value of 1.44 mm; range 0.81–2.58 mm) than in mandibular reconstructions (mean value of 1.33 mm; range 0.73–1.97 mm).

The difference in RMS between test 1 and 2 was on average 0.69 mm (range 0.02–1.77 mm).

Symmetry analysis showed optimal aesthetic outcomes. In the maxillectomy model, mean postoperative RMS was 1.45 mm (range 0.96–1.75 mm), which compares well with a mean preoperative value of 1.00 mm (range 0.62–1.31 mm). Concerning mandibulectomies, the difference between pre- and postoperative RMS was only slightly higher. In fact, the mean postoperative RMS was 1.90 mm (range 1.12–3.43 mm) compared to a preoperative value of 1.22 mm (range 1.07–1.65 mm).

#### Occlusal morphological conformance

We conducted this analysis on two dentulous specimens and four edentulous specimens.

In the entire series, the mean RMS was 1.53 mm (range 0.91–2.85 mm). Results were slightly better in mandibular reconstructions (mean, 1.40 mm; range 1.14–1.68 mm) compared to maxillectomies (mean, 1.66 mm; range 0.91–2.85 mm).

In dentate specimens, the overall mean RMN was 1.26 mm. RMS of maxillary reconstructions were 1.14 mm (type I defect) and 0.91 mm (type III defect), whereas for the mandibular reconstruction they were 1.68 mm (type I defect) and 1.31 mm (type II defect).

#### Mechanical stress test

Maxillary scaffolds were able to tolerate a mean force equal to 318.60 N (range 257 N [type III defect] and 411 N [type I defect]).

The mandibular scaffolds with designed holes tolerated a force between 205 N (type III defect) and 429 N (type I defect), with a mean value of 286.40 N. Scaffolds with designed supports for fixation were able to tolerate a force greater than 450 N in all cases (6 of 6 cases). Ruptures never occurred at the level of the scaffolds or fixation systems, but always in the surrounding native bone (12 of 12 cases).

## Discussion

Our data support the feasibility and reproducibility of a reconstructive algorithm for complex maxillary and mandibular defects which includes the use of customized, CAD 3D-printed polymeric scaffolds. The production process was straightforward, accurate, and efficient. Production time (about 2 days) is more than acceptable in a clinical scenario. Segmentation and scaffold design could be easily performed by medical personnel, which is relevant for the definition of cutting lines and extent of resection. Post-segmentation modifications were carried out in collaboration with engineers. Quality assessment of the production process was satisfactory overall. In particular, bone morphological conformance and symmetry were optimal, and the support for mandibular fixation (in place of the titanium plates) never broke. Recently, similar production algorithms have been investigated in preclinical setting for complex head and neck reconstruction, such as the nasal pyramid [19] and the auricle [20].

Specific tests on the polymeric material used to print the scaffolds were out of the scope of the present paper. We selected PLA because it is FDA approved, has osteoinductive and osteoconductive properties [21], and is suitable for widespread used printing technologies, such as FFF. Further research by our group on this reconstructive algorithm will address the possibility of bioengineering the scaffold with hMSC [22, 23]; in that context, the choice of the material will be paramount to guarantee both mechanical solidity and cells growth and viability.

### The surgical algorithm for scaffold in-setting and fixation

In our algorithm, the role of the navigation system was paramount to obtain excellent correspondence between the predicted and real osteotomies, and optimize the matching between the surfaces of the scaffold and the bony edges, which is recognized as a key factor to allow new bone formation [24]. In our tests, the navigation system set-up was optimal in all cases (systematic error always < 1 mm); accordingly, the scaffold was always easily plugged into the defect and its surface perfectly faced the bony boundaries without any need for drilling to adjust the shape. This finding is in accordance with a preclinical study from our group on the use of 3D-printed scaffolds to reconstruct complex clival defects and highlight the necessity of a meticulous setting of the navigation system in a hypothetical clinical scenario [25].

Scaffold fixation was intuitive and timesaving. Interestingly, mandibular supports for fixation made the process much quicker and never broke either during driving of the screws or stress tests. Therefore, it is a promising technique to avoid the use of titanium plates, which are at risk of both extrusion and infection. In case of complex maxillary defects (type III), the positioning of a screw in the root of the pterygoid (if neither resected nor too pneumatized) can provide stability for the posterior part of the reconstruction. Even in this case, the use of the navigation system was essential to check for length and trajectory of the screw and avoid injury to the internal carotid artery.

### Reconstruction assessment provided promising functional and aesthetic outcomes independently from the complexity of the defect

Morphological conformances showed RMS values consistently below 3 mm (on average, in between 1.3 and 1.5 mm), which is considered optimal in the pertinent literature [14–16, 26]. Reproducibility was apparently good, although it should be noticed that every procedure was repeated only twice.

Mechanical stress tests were successful, with maximal force values that were in line with the average human bite, ranging from 325 to 421 N [27–29]. However, these results should be weighed cautiously, since the human bite requires the action of different powerful muscles that act along different vectors of force, which is impossible to recreate in a cadaver head. Our stress test was aimed at measuring the maximal vertical force carried by the reconstruction before breaking and could be only a faded surrogate of a human bite. It was mostly addressed to test the mechanical resistance of the fixation systems, which was overall satisfactory as the site of rupture never included this site.

With the aim of testing the efficacy of the algorithm in different scenarios, we analyzed three types of maxillary and mandibular defects with increasing degree of complexity from a reconstructive standpoint. Interestingly, our findings are apparently independent of the complexity of the defect (Table 1). This is relevant and supports the efficacy and applicability of this reconstructive protocol in different clinical scenarios.

Restoration of the dentition was an exploratory test to verify the accuracy of the production process in such a demanding setting. In an ideal clinical scenario, the production of a dentate scaffold would allow a definitive single-stage reconstruction. Overall, the task confirmed its complexity and made all the production process more cumbersome. In the future, more sophisticated protocols of scaffold planning and printing and different materials for teeth need to be investigated.

### Limitations of the study

Our study has some limitations. First, the limited sample size prevented any statistical analysis, and the reconstruction algorithm could be tested only twice for each type of defect, which is the minimum to verify reproducibility. Then, we applied our protocol only in a preclinical setting on cadaver heads. This limits the validity of mechanical stress tests and precludes further analysis concerning the risk for infections, evaluation of new bone formation, and long-term integration of the scaffold.

### Future directions

As previously pointed out, the present study is intended as a preliminary feasibility analysis of a wider research line including bioengineering of the scaffold with hMSC. Therefore, the current algorithm is probably not applicable in clinics as it is.

Two relevant issues must be addressed: the protection from infections and the boost of scaffold integration.

The first one encompasses the resurfacing of the scaffold with vascularized tissue to avoid exposure to contaminated regions such as the oral, nasal, and pharyngeal cavities. In our study, this goal was attempted only for maxillary scaffolds using regional pedicled mucosal or fascial flaps. Coverage of mandibular plugs could be achieved by surrounding soft tissue in case of exclusive bony ablation, or by the fascio-cutaneous flap already used to restore larger soft tissue resections.

An alternative option could be the planning of a bi-phasic scaffold, with a rigid, resorbable core contoured by a cell-friendly bioactive shell to speed up mucosal coverage [30].

Scaffold integration has been investigated in animal models, such as rabbit [7, 20, 31] and dog [32] with uneventful follow-up and evidence of progressive mineralization of the scaffold. Han and coworkers described a small series of 3 patients with limited maxillary defects successfully repaired with patient-specific, CAD and 3D-printed polycaprolactone scaffolds that were inserted into the defect to recreate volume and achieve symmetry [33]. However, in case of extensive bony defects as the ones considered in the present study, scaffold integration through spontaneous bony repair is not conceivable. The bioengineering of the scaffold with hMSC is meant to speed up new bone formation, which ideally should replace in a timely manner the resorbable scaffold and recreate the ablated bone, providing a complete functional and aesthetic restoration of the defect. Preliminary results from our groups are promising [22, 23], although new tests in larger animal models are mandatory. In literature, so far several studies have investigated scaffold bioengineering with mesenchymal stem cells to propel new bone or cartilage formation [7, 34–38]. Some attempts on large animals have yielded encouraging insights that bioengineered scaffolds also work in large size defects [39, 40].

## Conclusion

Reconstruction of complex maxillary and mandibular defects using customized, CAD and 3D-printed polymeric scaffolds was feasible and reproducible in a preclinical setting. The quality of the reconstruction was high both in terms of functional and aesthetic outcomes. Cooperation among medical personnel and engineers is essential to achieve optimal results. Bioengineering of the scaffold to boost new bone formation and preclinical animal studies are warranted to translate this protocol into real-world practice.

### Supplementary Information

Below is the link to the electronic supplementary material.

Supplementary file1Supplementary fig. 1. Post-segmentation modifications of the scaffold for a type II mandibular defect. A, design of the integrated supports and holes for scaffold fixation with screws; B, corresponding 3D-printed scaffold (TIF 87 KB)

Supplementary file2Supplementary fig. 2. Setting of the surgical simulation and definition of the planned osteotomies using the GTx-Eyes 3D navigation system (TIF 182 KB)

Supplementary file3Supplementary fig. 3. Bone conformational analysis. A, the blue and gray surfaces were obtained from pre- and postoperative CT DICOM, respectively. B, the spatial difference between the two surfaces is expressed in mm through the root mean square deviation (red circle). The green areas represent the areas where the discrepancy is less than 1 mm; in this case they are equal to 61% of the total surface (blue arrow) (TIF 100 KB)

## Data Availability

Data of the study are available upon request.

## Abbreviations

3D: Three-dimensional
AM: Additive manufacturing
CAD: Computer-aided design
CT: Computed tomography
FDA: Food and drug administration
FFF: Fused Filament Fabrication
PLA: Polylactic acid
RMS: Root mean square
